# The preventative role of exogenous melatonin administration to patients with advanced cancer who are at risk of delirium: study protocol for a randomized controlled trial

**DOI:** 10.1186/s13063-016-1525-8

**Published:** 2016-08-11

**Authors:** Shirley Harvey Bush, Nathalie Lacaze-Masmonteil, Marie Theresa McNamara-Kilian, Alistair Richard MacDonald, Sallyanne Tierney, Franco Momoli, Meera Agar, David Christopher Currow, Peter Gerard Lawlor

**Affiliations:** 1 Division of Palliative Care, Department of Medicine, University of Ottawa, 43 Bruyère Street, Ottawa, ON K1N 5C8 Canada; 2 Bruyère Research Institute (BRI), 43 Bruyère Street, Ottawa, ON K1N 5C8 Canada; 3 Ottawa Hospital Research Institute (OHRI), 501 Smyth Rd, Ottawa, ON K1H 8L6 Canada; 4 Bruyère Continuing Care, 43 Bruyère Street, Ottawa, ON K1N 5C8 Canada; 5 Children’s Hospital of Eastern Ontario Research Institute, 401 Smyth Road, Ottawa, ON K1H 5B2 Canada; 6 School of Epidemiology, Public Health and Preventive Medicine, University of Ottawa, Centre for Practice-Changing Research (CPCR), 501 Smyth Road, Room L1231, Box 201B, Ottawa, Ontario K1H 8L6 Canada; 7 Centre of Cardiovascular and Chronic Care, Faculty of Health, University of Technology Sydney, Level 3, 235 Jones Street, Ultimo, NSW 2007 Australia; 8 Discipline, Palliative and Supportive Services, Bedford Park, Flinders University, Adelaide, SA Australia

**Keywords:** Melatonin, Delirium, Advanced cancer, Feasibility study, Randomized controlled trial (RCT), Sleep, Pilot, Prevention

## Abstract

**Background:**

Delirium is a very common and distressing neuropsychiatric syndrome in palliative care. Increasing age, the presence of dementia and advanced cancer are well-known predisposing risk factors for delirium development. Sleep-wake cycle disturbance is frequently seen during delirium and melatonin has a pivotal role in the regulation of circadian rhythms. Current evidence across various settings suggests a potential preventative role for melatonin in patients at risk of delirium, but no studies are currently reported in patients with advanced cancer. The aim of this article is to describe the design of a feasibility study that is being conducted to inform a larger randomized, placebo-controlled, double-blind trial (RCT) to evaluate the role of exogenously administered melatonin in preventing delirium in patients with advanced cancer.

**Methods/Design:**

Adult patients with a cancer diagnosis who are admitted to the palliative care unit will be randomized into a treatment or placebo group. The pharmacological intervention consists of a single daily dose of immediate-release melatonin (3 mg) at 21:00 ± 1 h, from day 1 to day 28 of admission. The primary objective of this initial study is to assess the feasibility of conducting the proposed RCT by testing recruitment and retention rates, appropriateness of study outcome measures, acceptability of study procedures and effectiveness of the blinding process. The primary outcome measure of the proposed larger RCT is time to first inpatient incident episode of delirium. We also plan to collect data on incident rates of delirium and patient-days of delirium, adjusting for length of admission.

**Discussion:**

The outcomes of this feasibility study will provide information on recruitment and retention rates, protocol violation frequency, effectiveness of the blinding process, acceptability of the study procedures, and safety of the proposed intervention. This will inform the design of a fully powered randomized controlled trial to evaluate the preventative role of melatonin administration in patients with advanced cancer.

**Trial registration:**

Registered with ClinicalTrials.gov: NCT02200172 Registered on 21 July 2014.

Health Canada protocol number: BRI-MELAT-2013 (Final approved protocol version (Version 3): 18 June 2014) (Notice of Amended Authorization (NOA) received 14 November 2014).

**Electronic supplementary material:**

The online version of this article (doi:10.1186/s13063-016-1525-8) contains supplementary material, which is available to authorized users.

## Background

Delirium is a very common and distressing neuropsychiatric syndrome for patients and their families in palliative care settings [1–3]. Delirium occurrence rates of over 80 % have been reported in the last hours and days before death [4, 5]. Conducting research in this population is challenging and, not surprisingly literature, data are limited owing to patient frailty and high attrition rates in association with the context of advanced disease [6–8].

Delirium is characterized by altered awareness, attention and cognitive deficits, perceptual disturbances, and a fluctuating course with potential intervals of relative lucidity that likely contribute to its underdiagnosis [9]. Delirium impairs patient communication, thus challenging the assessment of pain and other symptoms [1]. It often occurs at a critical juncture in advanced disease, when a preexisting narrow window of opportunity to communicate with family may be further compromised.

Delirium is associated with increases in morbidity, mortality, health care costs and, most importantly, in levels of patient and family distress [10–17]. Increasing age and the presence of dementia are well-known predisposing risk factors for the development of delirium [18]. Given the increasing elderly proportion of the population, and that cancer is predominantly a disease of the elderly, there is a fundamental need to develop primary, secondary and tertiary preventative strategies for delirium in these patients [19, 20]. Using early prophylactic strategies may reduce incident delirium, thereby reducing patient morbidity and mortality, [21] and suffering [22].

Although a multicomponent intervention study in a geriatric setting resulted in a 34 % reduction in delirium incidence, [23] a less elaborate intervention strategy that targeted cancer patients in palliative care settings failed to demonstrate a reduction in delirium incidence, though many study limitations were noted [22]. The recently published National Institute for Health and Clinical Excellence (NICE) guideline on the diagnosis, prevention and management of delirium concluded that delirium prevention would be cost-effective and that approximately 30 % of all episodes of delirium could be prevented through a multicomponent package of basic care interventions, many of a nonpharmacological nature [18]. A recent Cochrane review affirmed the urgent need for well-designed trials of delirium prevention [24, 25]. In 2010, the Canadian Coalition for Seniors’ Mental Health published “Guidelines on the assessment and treatment of delirium in older adults at the end of life” [26]. Prior to conducting a larger preventative trial, we plan to implement these guidelines on our palliative care unit in tandem with a feasibility study to examine the role of melatonin in the prevention of delirium.

Although the phenomenon of sleep-wake cycle disturbance is not a core diagnostic criterion for delirium, its prevalence has been reported in the 75–100 % range in studies of delirium in cancer patients [27, 28]. This phenomenon most likely reflects a circadian rhythm disturbance [29–31]. Melatonin (*N*-acetyl-5-methoxytryptamine) is a neuro-hormone that is derived sequentially from tryptophan and serotonin. Two enzymes in the pineal gland subsequently catalyze the synthesis of melatonin from serotonin [32]. Melatonin has a pivotal role in the regulation and synchronization of the sleep-wake cycle and circadian rhythms. Melatonin has immune-enhancing, anti-inflammatory and anticachectic effects, in addition to antioxidant and oncostatic properties [33–36]. It is secreted in response to retinal photoreceptors releasing norepinephrine with the onset of darkness. Endogenous plasma melatonin levels begin to rise around 22:00 h at night, peak around 03:00 h at approximately 100 pg/ml, before dropping back to usual low daytime levels by 09:00 h [37]. Endogenous peak plasma melatonin levels vary greatly between individuals and are reduced with age, increased cognitive impairment and in critical illness [37–39]. Melatonin is rapidly metabolized, mainly in the liver, by hydroxylation. Following subsequent conjugation with sulphuric or glucuronic acid, it is excreted in the urine [32, 40]. The chief metabolite of melatonin, 6-sulfatoxymelatonin (6-SMT), closely parallels serum melatonin concentrations in healthy individuals [32, 37, 40]. The role of melatonin in the pathogenesis of delirium has been hypothesized in terms of a deficient state and sleep dysregulation [41–43].

Melatonin supplementation has been suggested as a preventative measure for patients at risk of postoperative delirium [44, 45]. Further interest in this role of prophylactic exogenous melatonin treatment has been prompted by a 2010 systematic review, which concluded that in addition to melatonin having benefit in the reduction of “sundowning” or agitated behavior in dementia patients, it could have the same positive effects in patients with delirium [29]. More recently, a randomized, placebo-controlled, double-blind trial in 145 elderly internal medicine inpatients in London, Ontario reported a reduced risk of delirium in the low-dose (0.5 mg) melatonin-treated group versus placebo, reflected by incidence rates of 12 % versus 31 %, respectively [46]. A subgroup analysis in a recent meta-analysis concluded that melatonin supplementation reduced the incidence of delirium by 75 % in hospitalized medical patients of 65 years of age or older [47]. Melatonin appears to be well-tolerated and few serious side effects have been reported [48–50]. We hypothesize that melatonin may have a preventative role in the management of delirium in the palliative care population. Given the absence of data on its use in this population and the mixed results in other populations, we plan to test our hypothesis in a randomized controlled trial (RCT). The primary outcome measure of the proposed RCT is time to first inpatient incident episode of delirium. We also plan to collect data on incident rates of delirium and patient-days of delirium, adjusting for length of admission.

The challenges of conducting RCTs in palliative care settings have been well-documented [51]. Hagen et al. make a strong case for conducting an initial formal feasibility study prior to a RCT: this allows researchers to test the multiple dimensions of feasibility such as recruitment, retention and acceptability of study procedures [51]. This paper describes the design of a pilot and feasibility study to examine the preventative role of exogenous melatonin administration in patients with advanced cancer who are at risk of delirium. We chose to use an immediate-release (IR) preparation of melatonin as this formulation may have the advantage of providing a short-lived higher peak concentration of melatonin when compared to slow-release preparations [52]. In addition, an IR preparation would appear to better replicate the natural circadian pattern of in-vivo melatonin secretion and thus facilitate its potential regulatory role in homeostasis.

There is at least some degree of semantic ambiguity in the literature regarding the distinction between feasibility and pilot studies [53]. Given that our feasibility study will represent a miniature version of the larger randomized trial, we therefore also view this feasibility study as a pilot study. For consistency of reference, we will mainly use the term feasibility study in this publication.

### Primary objective of the feasibility study

To assess the feasibility of conducting a proposed RCT by determining (1) recruitment rates and retention rates, (2) the appropriateness of the main study outcome measures as indicated by protocol violation frequency, and some preliminary data on time to onset of first episode of incident delirium during their inpatient admission, (3) the acceptability of the study procedures to patients, families and palliative care unit staff including pharmacy, and (4) the effectiveness of the blinding process.

### Secondary objectives of the feasibility study

1. To make necessary adjustments to the main study protocol and assess subsequent feasibility in an iterative manner. This includes a feasibility assessment of the data collection process in relation to the predisposing and precipitating risk factors for delirium
2. To facilitate the concomitant implementation of standard delirium prevention and management guidelines on the palliative care unit prior to proceeding with the main RCT
3. To assess the safety of the proposed intervention in this population

## Methods/Design

### Study setting

This study is being conducted on the 31-bed inpatient palliative care unit (PCU) at Élisabeth Bruyère Hospital, a university teaching unit at Bruyère Continuing Care in Ottawa, Ontario, Canada. The study is sponsored by Bruyère Research Institute.

### Study design

**Fig. 1:**
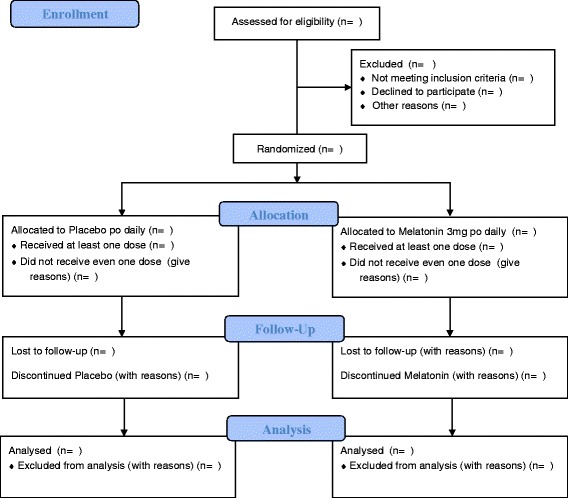
Overview of study design for melatonin feasibility study

This investigator-initiated feasibility study is a randomized, placebo-controlled, double-blind, single-center trial of a daily administered single dose of melatonin administered per os to prevent delirium in patients with advanced cancer. It is being conducted in order to inform a larger randomized, placebo-controlled, double-blind, parallel-group, multicenter trial. Figure 1 shows the Consolidated Standards of Reporting Trials (CONSORT) overview of the larger RCT that will be piloted in our feasibility study. A populated Standard Protocol Items: Recommendations for Interventional Trials (SPIRIT) checklist for this manuscript is also provided (see Additional file 1).

### Sample size

There are varying viewpoints regarding the appropriate sample size for feasibility and pilot studies [54]. Whereas estimation may be guided on the basis of confidence intervals and standard deviation for continuous outcome measures, the situation is less clear in the case of pilot studies with time to event measures, such as time to onset of first incident episode of delirium. In inpatient palliative care settings, delirium incidence rates have been reported in the 3–45 % range [55]. In our main RCT, we anticipate a delirium incidence rate of approximately 25 % in the placebo arm by day 28 of admission. This would result in a 75 % administrative censoring proportion in the larger study. In addition, we arbitrarily set the anticipated proportion of noninformative censoring due to withdrawal or loss to follow-up at 10 %. We currently estimate a sample size requirement of *N* = 410 for the main study to detect an effect size (hazard ratio) of 0.5, given an alpha level of 0.05 and power of 80 %, using STATA version 14.1 (StataCorp LP, College Station, TX, USA) [56]. We further estimate that a sample size of 60 (arbitrarily set at approximately 15 % of the currently projected sample size of the larger study) with 30 participants in each arm will be adequate to provide useful data regarding the feasibility outcome measures.

### Recruitment and consent

Potential participants will be approached within 72 h of admission to the PCU. An assessment will be made by the attending physician as to their capacity to understand the risks and benefits of participation in a trial. A member of the primary “circle of care” will ask potential participants or their substitute decision-maker (SDM) for their verbal consent to be contacted by an authorized research team member. Following this initial consent, potential participants or their SDM will be approached by a clinical research nurse (CRN) or a clinical research assistant (CRA) and be provided with written and oral information about the study, including risks and benefits. If a participant or their SDM chooses to enroll, signed informed consent is obtained before commencing the study.

### Eligibility criteria

The study population consists of patients of 18 years and older with advanced cancer who are admitted to the PCU. Additional inclusion criteria are the ability to speak English, a Palliative Performance Scale (PPS) score [57] of 30 % or higher at the time of consent, and cognitive capacity to provide informed consent (or an accessible SDM who is able to provide consent). Exclusion criteria are: delirium present on admission, as assessed clinically with the CAM [58]; known psychotic disorder other than dementia; inability to take medications sublingually or via gastrostomy tube; known allergy to melatonin or placebo content; use of melatonin within the 2 weeks preceding admission; patients on warfarin treatment or other anticoagulant administered per os, on other investigational agents or treatments or on immunosuppressant medication in the context of autoimmune disease or post organ transplantation; communication problems that cannot be accommodated, including deafness, tracheostomy, aphasia, dysarthria or emotional distress; severe visual impairment or being designated legally blind; and pregnancy or lactation.

### Randomization, allocation concealment and blinding

This study aims to minimize selection bias by using randomization and allocation concealment. Upon confirmation of eligibility, the participant will be randomized in a 1:1 ratio of melatonin to placebo treatment. We will use an independent web-based randomization system (Ottawa Hospital Research Institute (OHRI) Data Management Services) that provides the group allocation according to a randomization list, pregenerated by an independent statistician. This central randomization system ensures allocation concealment. The randomization master list will be kept confidential and secure by OHRI Data Management Services, the Director of Pharmacy at Bruyère Continuing Care and the pharmacy technician who sets up the prepackaged study (active or placebo) medication. The confidential participant assignment record is held by the Director of Pharmacy. The study investigators, other research team members, PCU pharmacist, PCU physicians, PCU nurses, dispensing pharmacist and technician and other health care personnel will remain blinded to the study medication, except in the case of approved emergency unblinding.

### Study drug and intervention protocol

The study medication tablets for both melatonin and placebo appear identical and are prepackaged in small blue containers labeled according to Natural Health Products Regulations and Good Labeling Practices. Both melatonin and matching placebo tablets have been manufactured and obtained from the same pharmaceutical company. Once prescribed by one of the PCU physicians, the dispensed container is sent to the unit by a pharmacy technician.

On study day 1 (D1), enrolled patients receive the study medication consisting of a tablet administered sublingually or via gastrostomy tube of either 3 mg melatonin (immediate-release) or placebo at 21:00 h (±1 h) until study day 28 (D28) of informed consent, or earlier in the event of death or discharge. In the event of incident delirium occurring before D28, the study drug is discontinued immediately. As standard practice on our unit, patients are assessed at the end of each 8-hour nursing shift with the Nursing Delirium Screening Scale (Nu-DESC) [59] to observationally screen for the presence of delirium. The Confusion Assessment Method (CAM) [58] is subsequently performed by a PCU physician as a confirmatory diagnostic test of delirium if Nu-DESC screening is positive.

### Withdrawals and discontinuation

The study medication will be stopped if any of the following occurs:

- Participant death or discharge
- Incident delirium
- Participant experiences adverse event(s) that require discontinuation in the judgment of the principal investigator or designee
- Participant has a need for additional medication that would interfere with the trial
- The participant neglects to follow trial instructions
- The participant or their legally authorized representative request consent withdrawal
- The sponsor or the principal investigator or the Data Safety Monitoring Board (DSMB) or a government agency, such as Health Canada, terminates the study

### Data collection

#### Data recorded at admission

Table 1 summarizes the study schedule of enrollment, interventions and assessments using the SPIRIT template [60]. Routine data will be recorded at admission and include: gender, age, documentation of cancer diagnosis and current medication list. Routine assessments at admission to be recorded include: the PPS [57], the CAM [58], the Short Orientation-Memory-Concentration Test (SOMCT) [61], the Edmonton Classification System for Cancer Pain (ECS-CP) [62] and the Edmonton Symptom Assessment System-revised (ESAS-r) [63]. These evaluations are routinely conducted on all patients on admission to the PCU as part of the admission process. Routine screening with the Nu-DESC [59] starts from the time of patient admission.

**Table 1:** Schedule of enrollment, interventions and assessments (using the SPIRIT template [60]) for the melatonin feasibility study

	Study period								
	Enrollment	Allocation	Post-allocation						Close-out
Timepoint	At admission	Within 72 h of admission	Day 1 to Day 28	Day 29 and Day 30	At suspicion of delirium	Delirium diagnosis confirmed	24 (16–32) h after delirium diagnosis	Within 3 days after delirium diagnosis	30-day period after stop of trial product
			(D1–D28)	(D29 + 30)					
Enrollment:									
Eligibility screen: verbal consent to be contacted by research team member	X								
Informed consent		X							
Allocation		X							
Interventions:									
Administer melatonin/placebo			X Daily: @ 21:00 ± 1 h			Discontinue trial product (trial product return/count)			
Assessments:									
Medical history	X								
GOC	X					X			
PPS	X								
ECS-CP	X								
CAM	X				X				
SOMCT	X								
Nu-DESC	X		X						
ESAS-r	X		X						
Concomitant medications/NHP	X		X						
AE data collection		X	X	X	X	X	X	X	
BRP-DICT			X D1 only						
CCI			X D1 only						
ISI			X D1, 14, 28 ± 2 days						
PCU physician CGR						X			
MDAS							X		
PP-DICT								X	
Contact HCP for SAE outcome/death									X

Abbreviations: *AE/SAE* adverse event/serious adverse event, *BRP-DICT* Baseline Risk Profile for Delirium in the Cancer Trajectory, *CAM* Confusion Assessment Method, *CCI* Charlson Co-morbidity Index, *CGR* Clinical Global Rating Scale, *ESAS-r* Edmonton Symptom Assessment System-revised, *GOC* goals of care, *HCP* health care professional, *ISI* Insomnia Severity Index, *MDAS* Memorial Delirium Assessment Scale, *NHP* Natural Health Product, *Nu-DESC* Nursing Delirium Screening Scale, *PCU* palliative care unit, *PP-DICT* Precipitant Profile for Delirium in the Cancer Trajectory, *PPS* Palliative Performance Scale, *SOMCT* Short Orientation Memory Concentration Test

The PPS assesses functional status and measures progressive decline in palliative care patients. It is routinely completed within the first 24 h of admission to the PCU. The CAM is used for delirium screening at admission and has a diagnostic algorithm. (Nu-DESC ratings are not available at admission). The SOMCT is a brief, validated cognitive screening tool. The ECS-CP generates a complexity profile in relation to the challenge of achieving stable cancer pain control. The ESAS-r is a widely used tool to describe symptom intensity for nine common symptoms (pain, tiredness, drowsiness, nausea, appetite, shortness of breath, anxiety, depression and wellbeing) in palliative care, and is based on self-report. The Nu-DESC is a brief, validated observational screening tool for delirium that is completed by the patient’s bedside nurse at the end of each 8-hour nursing shift.

#### Data recorded during study

Data relating to predisposing factors for delirium will be collected on study day 1 (D1) by the CRN or CRA using a standard checklist. The Charlson Co-morbidity Index (CCI) [64] and Baseline Risk Profile for Delirium in the Cancer Trajectory (BRP-DICT) (developed ad hoc by the senior author) will be rated on D1. The CRN or CRA will visit the PCU daily (on weekdays) to collect data on Nu-DESC and ESAS-r scores, CAM results and concomitant medications and enquire about adverse events while the participant is enrolled in the trial and up to 48 h after the trial medication has stopped. The designated goals of care (GOC), established by consensus following discussions with the patient or SDM, will be recorded on D1 and at the time of diagnosis of incident delirium. Consistent with patient and family wishes, the designated goals of care (ranging in category from full resuscitation to comfort care only) help to determine the degree of intensity applied to the investigation and treatment of an episode of delirium. This approach is consistent with the “Canadian guidelines on the assessment and treatment of delirium in older adults at the end of life” [26]. The Insomnia Severity Index (ISI) [65] is a short, subjective and validated tool that measures a patient’s self-reported views about his or her insomnia. This instrument will be rated by the patient on D1, and once in each of the time ranges D14 ± 2 d and D28 ± 2 d. The Memorial Delirium Assessment Scale (MDAS) [66] is a validated delirium-severity rating tool that also captures psychomotor and sleep-wake cycle disturbance. The MDAS will be rated by the CRN on weekdays in all incident cases of delirium encompassing the first 24 h of delirium. (It is not possible as part of this feasibility study to rate the MDAS on the weekends due to limited resources). The PCU attending physician will be independently asked to provide a Clinician Global Rating (CGR) of delirium severity at the time of delirium diagnosis. This rating has three categories: mild, moderate and severe. In addition, precipitating factors at the time of incident delirium will be collected by the CRN or CRA using a standard checklist (Precipitant Profile for Delirium in the Cancer Trajectory (PP-DICT)) developed ad hoc by the senior author. Table 1 summarizes the study schedule of enrollment, interventions and assessments.

### Data and Safety Monitoring

Adverse events will be graded by the investigator using the National Cancer Institute’s “Common terminology criteria for adverse events (NCI-CTCAE v 4.03)” [67]. Information about all adverse events, whether volunteered by the study participant, discovered by the investigator or designee’s questioning, laboratory test or other means, will be recorded and followed as appropriate. The Data and Safety Monitoring Board (DSMB) for this study will comprise one statistician methodologist, one palliative care specialist, and one individual knowledgeable in Natural Health Products for conduct monitoring and supervision of the clinical trial. The DSMB will meet at least once before the study starts and then meet according to the DSMB terms of reference. After every 20th enrolled patient, a trial monitor, who is not part of the research team, will review the trial documentation to ensure that the trial is being conducted according to the trial protocol, the International Conference on Harmonization Good Clinical Practice (ICH GCP), and regulatory requirements.

### Data analysis plan

This feasibility study is not powered to test efficacy. However, we anticipate that it will generate useful data regarding recruitment and retention rates, protocol violation frequency, and some preliminary data on time to onset of first episode of incident delirium during their inpatient admission. These data will further assist in the estimation of sample size for the larger study, as well as its projected duration and cost. Data will be reported regarding the acceptability of the study procedures to patients, families and palliative care unit staff, including pharmacy. The effectiveness of the blinding process will be assessed. Collectively, these outcome data will ultimately determine the feasibility of the larger trial. Although the statistical analysis in this feasibility study will be mainly descriptive, we will also assess time to first episode of incident delirium with a Kaplan-Meier survival analysis. The upper one-sided 95 % limit of median time to onset will also be derived to further guide sample size considerations for the full randomized trial.

## Discussion

We have chosen to limit the study participants to those with a cancer diagnosis, as a heterogeneous study sample with a variety of life-threatening illnesses could conceivably make it more difficult to achieve a good balance in the two study arms and thus lead to confounding. Our study will include patients with dementia, which is one of the strongest predisposing risk factor for the development of delirium, along with advanced age. Although this will generate some challenges in the assessment of delirium superimposed on dementia, we postulate that those with dementia may well be the group that is most likely to benefit from melatonin.

We have chosen to implement the “Canadian guidelines on the assessment and treatment of delirium in older adults at the end of life” [26] as part of this feasibility study. We do not anticipate that this will interfere with any data analysis, as the implementation of these guidelines will be consistent for all randomized study patients and all inpatients on the PCU. As part of our feasibility study, we are piloting standardized checklists that were designed to comprehensively and systematically capture data regarding predisposing risk factors for delirium at admission (through the BRP-DICT form) and precipitating factors for delirium in the 24 h post delirium diagnosis (through the PP-DICT form).

The research nurse and research assistant are documenting field notes during this study, thus adding a qualitative component to this study. These “lessons learned” will provide additional insights into the potential barriers and feasibility of subsequent studies. The outcomes of this feasibility study will provide information on recruitment and retention rates, protocol violation frequency, effectiveness of the blinding process, acceptability of the study procedures and safety of the proposed intervention. This will inform the design of a fully powered RCT to evaluate the preventative role of melatonin administration in patients with advanced cancer.

## Trial status

The trial is currently recruiting patients.

## Abbreviations

BRP-DICT, Baseline Risk Profile for Delirium in the Cancer Trajectory (BRP-DICT); CAM, Confusion Assessment Method; CCI, Charlson Comorbidity Index; CGR, Clinician Global Rating; CRA, clinical research assistant; CRN, clinical research nurse; DSMB, Data and Safety Monitoring Board; ECS-CP, Edmonton Classification System for Cancer Pain; ESAS-r, Edmonton Symptom Assessment System-revised; GOC, goals of care; ICH GCP, International Conference on Harmonization Good Clinical Practice; IR, immediate release; ISI, Insomnia Severity Index; MDAS, Memorial Delirium Assessment Scale; NCI-CTCAE v 4.03, National Cancer Institute Common Terminology Criteria for Adverse Events version 4.03; NICE, National Institute for Health and Clinical Excellence; Nu-DESC, Nursing Delirium Screening Scale; OHRI, Ottawa Hospital Research Institute; PCU, palliative care unit; PHIPA, Personal Health Information Protection Act; PP-DICT, Precipitant Profile for Delirium in the Cancer Trajectory; PPS, Palliative Performance Scale; RCT, randomized controlled trial; SDM, substitute decision-maker; SOMCT, Short Orientation-Memory-Concentration Test; TCPS 2, Tri-Council Policy Statement version 2

## Additional file

Additional file 1: SPIRIT checklist for melatonin feasibility study. Populated SPIRIT checklist (Page numbers refer to location in manuscript). (PDF 85 kb)

## References

[1] Bruera E, Miller L, McCallion J, Macmillan K, Krefting L, Hanson J (1992). Cognitive failure in patients with terminal cancer: a prospective study. J Pain Symptom Manage.

[2] Lawlor PG, Bruera ED (2002). Delirium in patients with advanced cancer. Hematol Oncol Clin North Am.

[3] Pereira J, Hanson J, Bruera E (1997). The frequency and clinical course of cognitive impairment in patients with terminal cancer. Cancer.

[4] Bush SH, Bruera E (2009). The assessment and management of delirium in cancer patients. Oncologist.

[5] Massie MJ, Holland J, Glass E (1983). Delirium in terminally ill cancer patients. Am J Psychiatry.

[6] Lawlor PG, Gagnon B, Mancini IL, Pereira JL, Hanson J, Suarez-Almazor ME (2000). Occurrence, causes, and outcome of delirium in patients with advanced cancer: a prospective study. Arch Intern Med.

[7] Morita T, Tei Y, Tsunoda J, Inoue S, Chihara S (2001). Underlying pathologies and their associations with clinical features in terminal delirium of cancer patients. J Pain Symptom Manage.

[8] Leonard M, Agar M, Mason C, Lawlor P (2008). Delirium issues in palliative care settings. J Psychosom Res.

[9] American Psychiatric Association (2013). Diagnostic and statistical manual of mental disorders: DSM-5.

[10] Dasgupta M, Hillier LM (2010). Factors associated with prolonged delirium: a systematic review. Int Psychogeriatr.

[11] Pautex S, Herrmann FR, Zulian GB (2008). Factors associated with falls in patients with cancer hospitalized for palliative care. J Palliat Med.

[12] Siddiqi N, Holmes JD, House AO (2006). Occurrence and outcome of delirium in medical in-patients: a systematic literature review. Age Ageing.

[13] Balas MC, Happ MB, Yang W, Chelluri L, Richmond T (2009). Outcomes associated with delirium in older patients in surgical ICUs. Chest.

[14] Breitbart W, Gibson C, Tremblay A (2002). The delirium experience: delirium recall and delirium-related distress in hospitalized patients with cancer, their spouses/caregivers, and their nurses. Psychosomatics.

[15] Morita T, Hirai K, Sakaguchi Y, Tsuneto S, Shima Y (2004). Family-perceived distress from delirium-related symptoms of terminally ill cancer patients. Psychosomatics.

[16] Bruera E, Bush SH, Willey J, Paraskevopoulos T, Li Z, Palmer JL (2009). Impact of delirium and recall on the level of distress in patients with advanced cancer and their family caregivers. Cancer.

[17] Caraceni A, Nanni O, Maltoni M, Piva L, Indelli M, Arnoldi E (2000). Impact of delirium on the short term prognosis of advanced cancer patients. Italian Multicenter Study Group on Palliative Care. Cancer.

[18] O’Mahony R, Murthy L, Akunne A, Young J (2011). Synopsis of the National Institute for Health and Clinical Excellence guideline for prevention of delirium. Ann Intern Med.

[19] Agar M, Lawlor P (2008). Delirium in cancer patients: a focus on treatment-induced psychopathology. Curr Opin Oncol.

[20] Lawlor PG, Fainsinger RL, Bruera ED (2000). Delirium at the end of life: critical issues in clinical practice and research. JAMA.

[21] Bourne RS, Tahir TA, Borthwick M, Sampson EL (2008). Drug treatment of delirium: past, present and future. J Psychosom Res.

[22] Gagnon P, Allard P, Gagnon B, Merette C, Tardif F (2012). Delirium prevention in terminal cancer: assessment of a multicomponent intervention. Psychooncology.

[23] Inouye SK, Bogardus ST, Charpentier PA, Leo-Summers L, Acampora D, Holford TR (1999). A multicomponent intervention to prevent delirium in hospitalized older patients. N Engl J Med.

[24] Siddiqi N, Stockdale R, Britton AM, Holmes J (2007). Interventions for preventing delirium in hospitalised patients. Cochrane Database Syst Rev.

[25] Tabet N, Howard R (2009). Pharmacological treatment for the prevention of delirium: review of current evidence. Int J Geriatr Psychiatry.

[26] Canadian Coalition for Seniors’ Mental Health. Guideline on the assessment and treatment of delirium in older adults at the end of life. http://ccsmh.ca/projects/delirium/. Accessed 22 Jan 2016.

[27] Meagher DJ, Moran M, Raju B, Gibbons D, Donnelly S, Saunders J (2007). Phenomenology of delirium. Assessment of 100 adult cases using standardised measures. Br J Psychiatry.

[28] Bosisio M, Caraceni A, Grassi L (2006). Phenomenology of delirium in cancer patients, as described by the Memorial Delirium Assessment Scale (MDAS) and the Delirium Rating Scale (DRS). Psychosomatics.

[29] de Jonghe A, Korevaar JC, van Munster BC, de Rooij SE (2010). Effectiveness of melatonin treatment on circadian rhythm disturbances in dementia. Are there implications for delirium? A systematic review. Int J Geriatr Psychiatry.

[30] Meagher D (2009). Motor subtypes of delirium: past, present and future. Int Rev Psychiatry.

[31] Fitzgerald JM, Adamis D, Trzepacz PT, O’Regan N, Timmons S, Dunne C (2013). Delirium: a disturbance of circadian integrity?. Med Hypotheses.

[32] Brzezinski A (1997). Melatonin in humans. N Engl J Med.

[33] Maestroni GJ (1993). The immunoneuroendocrine role of melatonin. J Pineal Res.

[34] Srinivasan V, Spence DW, Pandi-Perumal SR, Trakht I, Cardinali DP (2008). Therapeutic actions of melatonin in cancer: possible mechanisms. Integr Cancer Ther.

[35] Lissoni P (2002). Is there a role for melatonin in supportive care?. Support Care Cancer.

[36] Mahmoud F, Sarhill N, Mazurczak MA (2005). The therapeutic application of melatonin in supportive care and palliative medicine. Am J Hosp Palliat Care.

[37] Bourne RS, Mills GH (2006). Melatonin: possible implications for the postoperative and critically ill patient. Intensive Care Med.

[38] Magri F, Sarra S, Cinchetti W, Guazzoni V, Fioravanti M, Cravello L (2004). Qualitative and quantitative changes of melatonin levels in physiological and pathological aging and in centenarians. J Pineal Res.

[39] Mistraletti G, Sabbatini G, Taverna M, Figini MA, Umbrello M, Magni P (2010). Pharmacokinetics of orally administered melatonin in critically ill patients. J Pineal Res.

[40] Balan S, Leibovitz A, Zila SO, Ruth M, Chana W, Yassica B (2003). The relation between the clinical subtypes of delirium and the urinary level of 6-SMT. J Neuropsychiatry Clin Neurosci.

[41] Maldonado JR (2013). Neuropathogenesis of delirium: review of current etiologic theories and common pathways. Am J Geriatr Psychiatry.

[42] de Rooij SE, van Munster BC (2013). Melatonin deficiency hypothesis in delirium: a synthesis of current evidence. Rejuvenation Res.

[43] Hatta K, Kishi Y, Wada K, Takeuchi T, Odawara T, Usui C (2014). Preventive effects of ramelteon on delirium: a randomized placebo-controlled trial. JAMA Psychiatry.

[44] Miyazaki T, Kuwano H, Kato H, Ando H, Kimura H, Inose T (2003). Correlation between serum melatonin circadian rhythm and intensive care unit psychosis after thoracic esophagectomy. Surgery.

[45] Olofsson K, Alling C, Lundberg D, Malmros C (2004). Abolished circadian rhythm of melatonin secretion in sedated and artificially ventilated intensive care patients. Acta Anaesthesiol Scand.

[46] Al-Aama T, Brymer C, Gutmanis I, Woolmore-Goodwin SM, Esbaugh J, Dasgupta M (2011). Melatonin decreases delirium in elderly patients: a randomized, placebo-controlled trial. Int J Geriatr Psychiatry.

[47] Chen S, Shi L, Liang F, Xu L, Desislava D, Wu Q et al. Exogenous melatonin for delirium prevention: a meta-analysis of randomized controlled trials. Mol Neurobiol. 2016;53(6):4046-53. doi:10.1007/s12035-015-9350-8. Epub 2015 Jul 21. 10.1007/s12035-015-9350-8 26189834

[48] Jansen SL, Forbes D, Duncan V, Morgan DG, Malouf R (2006). Melatonin for the treatment of dementia. Cochrane Database Syst Rev.

[49] Bjorvatn B, Pallesen S (2009). A practical approach to circadian rhythm sleep disorders. Sleep Med Rev.

[50] Karasek M (2007). Does melatonin play a role in aging processes?. J Physiol Pharmacol.

[51] Hagen NA, Biondo PD, Brasher PMA, Stiles CR (2011). Formal feasibility studies in palliative care: why they are important and how to conduct them. J Pain Symptom Manage.

[52] Herxheimer A, Petrie KJ (2002). Melatonin for the prevention and treatment of jet lag. Cochrane Database Syst Rev.

[53] Whitehead AL, Sully BG, Campbell MJ (2014). Pilot and feasibility studies: is there a difference from each other and from a randomised controlled trial?. Contemp Clin Trials.

[54] Thabane L, Ma J, Chu R, Cheng J, Ismaila A, Rios LP, et al. A tutorial on pilot studies: the what, why and how. BMC Med Res Methodol. 2010;10:1. doi:10.1186/1471-2288-10-1. 10.1186/1471-2288-10-1 PMC2824145 20053272

[55] Hosie A, Davidson PM, Agar M, Sanderson CR, Phillips J (2013). Delirium prevalence, incidence, and implications for screening in specialist palliative care inpatient settings: a systematic review. Palliat Med.

[56] STATA Data Analysis and Statistical Software. [14.1]. Texas: StataCorp LP; 2016. http://www.stata.com/

[57] Anderson F, Downing GM, Hill J, Casorso L, Lerch N (1996). Palliative performance scale (PPS): a new tool. J Palliat Care.

[58] Inouye SK, van Dyck CH, Alessi CA, Balkin S, Siegal AP, Horwitz RI (1990). Clarifying confusion: the confusion assessment method. A new method for detection of delirium. Ann Intern Med.

[59] Gagnon P, Allard P, Mâsse B, DeSerres M (2000). Delirium in terminal cancer: a prospective study using daily screening, early diagnosis, and continuous monitoring. J Pain Symptom Manage.

[60] Chan AW, Tetzlaff JM, Altman DG, Laupacis A, Gotzsche PC, Krleza-Jeric K (2013). SPIRIT 2013 statement: defining standard protocol items for clinical trials. Ann Intern Med.

[61] Katzman R, Brown T, Fuld P, Peck A, Schechter R, Schimmel H (1983). Validation of a short Orientation-Memory-Concentration Test of cognitive impairment. Am J Psychiatry.

[62] Fainsinger RL, Nekolaichuk C, Lawlor P, Hagen N, Bercovitch M, Fisch M (2010). An international multicentre validation study of a pain classification system for cancer patients. Eur J Cancer.

[63] Watanabe SM, Nekolaichuk C, Beaumont C, Johnson L, Myers J, Strasser F (2011). A multicenter study comparing two numerical versions of the Edmonton Symptom Assessment System in palliative care patients. J Pain Symptom Manage.

[64] Charlson ME, Pompei P, Ales KL, MacKenzie CR (1987). A new method of classifying prognostic comorbidity in longitudinal studies: development and validation. J Chronic Dis.

[65] Bastien C (2001). Validation of the Insomnia Severity Index as an outcome measure for insomnia research. Sleep Med.

[66] Breitbart W, Rosenfeld B, Roth A, Smith MJ, Cohen K, Passik S (1997). The Memorial Delirium Assessment Scale. J Pain Symptom Manage.

[67] U.S. Department of Health and Human Services. Common terminology criteria for adverse events (CTCAE). (v4.03: June 14, 2010). 2010. http://ctep.cancer.gov/protocolDevelopment/electronic_applications/ctc.htm. Accessed 22 Jan 2016.

